# Brain injury after 50 h of lung-protective mechanical ventilation in a preclinical model

**DOI:** 10.1038/s41598-021-84440-1

**Published:** 2021-03-03

**Authors:** Thiago G. Bassi, Elizabeth C. Rohrs, Karl C. Fernandez, Marlena Ornowska, Michelle Nicholas, Matt Gani, Doug Evans, Steven C. Reynolds

**Affiliations:** 1 Simon Fraser University, 8888 University Drive, Burnaby, BC V5A 1S6 Canada; 2 Lungpacer Medical, Inc, Suite 130, 601 W Cordova Street, Vancouver, BC V6B 1G1 Canada; 3 Fraser Health Authority, Royal Columbian Hospital, 260 Sherbrooke Street, New Westminster, BC V3L 3M2 Canada

**Keywords:** Neuroscience, Cell death in the nervous system, Neurogenesis, Neuroimmunology

## Abstract

Mechanical ventilation is the cornerstone of the Intensive Care Unit. However, it has been associated with many negative consequences. Recently, ventilator-induced brain injury has been reported in rodents under injurious ventilation settings. Our group wanted to explore the extent of brain injury after 50 h of mechanical ventilation, sedation and physical immobility, quantifying hippocampal apoptosis and inflammation, in a normal-lung porcine study. After 50 h of lung-protective mechanical ventilation, sedation and immobility, greater levels of hippocampal apoptosis and neuroinflammation were clearly observed in the mechanically ventilated group, in comparison to a never-ventilated group. Markers in the serum for astrocyte damage and neuronal damage were also higher in the mechanically ventilated group. Therefore, our study demonstrated that considerable hippocampal insult can be observed after 50 h of lung-protective mechanical ventilation, sedation and physical immobility.

## Introduction

The Intensive Care Unit (ICU) is the cornerstone of life support for critically ill patients^1^. Mechanical ventilation (MV) and sedation are elements of the treatment strategies commonly deployed in ICUs for the most profoundly ill patients^2^. These practices come with significant negative consequences, where both MV and sedation have been independently associated with neurological injury^3,4^. Preclinical work using high-tidal-volume MV, which is known to be injurious and is not used in standard practice, has shown a deleterious effect on the central nervous system (CNS)^5^. Additionally, drugs currently used for ICU sedation have been reported to be associated with neuronal damage^3,6^. However, it is unknown whether standard lung-protective MV in association with sedation is also associated with adverse neurological changes.

Quantification of brain injury has been based either on factors linked to the apoptotic cascade, or on the presence of neuroinflammatory cells^5,7^. Neurogenesis counterbalances cellular apoptosis and is influenced by inflammation^8^. Every day, approximately 700–1,400 new neurons are generated in the human hippocampus and about 9,000 in rodents^8,9^. The resilience of the hippocampus might be reflected in the turnover index^10^, which is calculated by subtracting the apoptotic index (proportion of apoptotic cells) from the mitotic index (proportion of new neurons). Many factors can interfere with the neurogenesis rate, but the effect of lung-protective MV in association with sedation on the generation of new neurons in the hippocampus is unknown.

We sought to explore neurological injury in a preclinical porcine model for 50 h, utilizing apoptosis, mitosis and inflammatory markers as indicators of brain injury. This might have significant translational importance in elucidating the factors leading to brain insult after critical illness^7^.

## Results

All the subjects studied were female with a median weight of 54 kg in the NV group and 57 kg in the MV group (p = 0.1147). All results are reported as medians with interquartile ranges unless otherwise specified.

### Fluid balance and spontaneous breathing efforts

Fluid balance delivered to the MV subjects was within the target range (0.1–2.0 ml/kg/day). Single spontaneous breaths during 50 h of lung-protective MV were detected on an average of eight episodes of per subject over the course of 36,917 breaths per experiment. No reverse triggering was noted.

### Hippocampal histological markers

**Figure 1:**
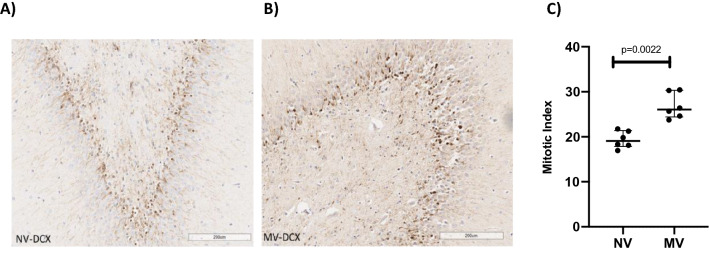
Slides of an NV subject’s hippocampus (**A**) and an MV subject’s hippocampus (**B**) after staining by doublecortin (DCX) assay, showing normal cells (blue) and doublecortin-positive cells (brown). Greater neurogenesis can be observed in the MV example than in the NV example. (**C**) Dot plot showing mitotic indices of 19.07 (17.84–21.38) in the NV group and 26.04 (24.41–30.34) in the MV group, p = 0.0022 (n = 6 per group).

**Figure 2:**
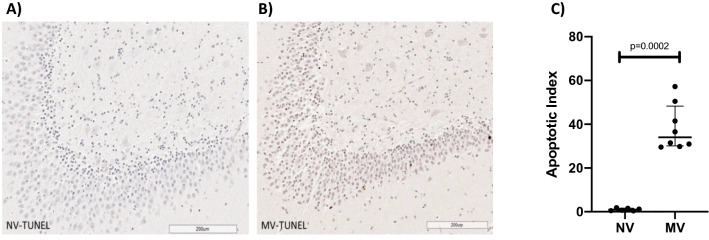
Slide of an NV subject’s hippocampus (**A**) and an MV subject’s hippocampus (**B**) after staining by TUNEL assay, showing normal cells (blue) and TUNEL-positive cells (brown). Considerably greater hippocampal cellular apoptosis can be observed in the MV example than in the NV example. (**C**) Dot plot showing apoptotic indices of 0.96 (0.50–1.61) in the NV group and 31.70 (29.79–43.76) in the MV group p = 0.0002 (n = 6–10 per group).

**Figure 3:**
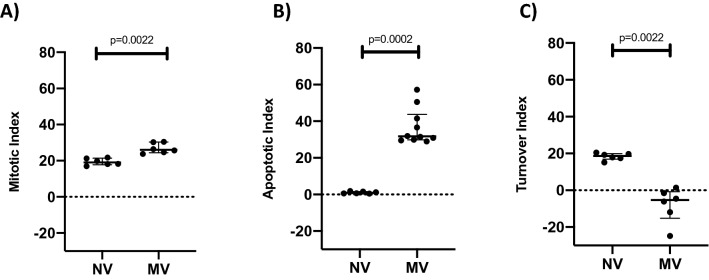
(**A**) Dot plot showing mitotic indices of 19.07 (17.84–21.38) in the NV group and 26.04 (24.42–30.34) in the MV group, p = 0.0022 (n = 6 per group). (**B**) Dot plot showing apoptotic indices of 0.96 (0.50–1.61) in the NV group and 31.70 (29.79–43.76) in the MV group p = 0.0002 (n = 6–10 per group). (**C**) Dot plot showing turnover indices of + 18.59 (16.86–19.92) in the NV group and − 5.32 (− 15.16- − 0.77) in the MV group, p = 0.0022 (n = 6 per group).

**Figure 4:**
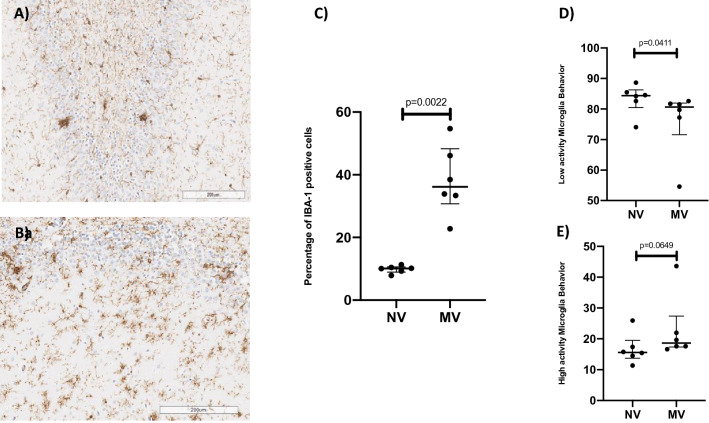
Slide of an NV subject’s hippocampus (**A**) and an MV subject’s hippocampus (**B**) after staining by IBA-1, showing normal cells (blue) and IBA-1-positive cells (brown). (**C**) Dot plot showing percentages of IBA-1-positive cells, combining the two behaviors of microglia, 10.12 (8.93–10.65) in the NV group and 36.17 (30.71–48.27) in the MV group, p = 0.0022 (n = 6 per group). (**D**) Dot plot showing percentages of low-activity microglia behavior, 84.40 (80.49–86.27) in the NV group and 80.63 (71.59–81.93) in the MV group, p = 0.0411 (n = 6 per group). (**E**) Dot plot showing percentages of high-activity microglia behavior, 15.60 (13.73–19.51) in the NV group and 18.63 (17.37–27.38) in the MV group, p = 0.0649 (n = 6 per group).

The mitotic index was significantly lower in the NV group (19.07; 17.84–21.38) than in the MV group (26.04; 24.41–30.34; p = 0.0022) (Fig. 1A–C). The apoptotic index was significantly lower in the NV group (0.96; 0.50–1.61) than in the MV group (31.70; 29.79–43.76; p = 0.0002) (Fig. 2A–C). The turnover index was significantly different between the groups, being positive in the NV group (+ 18.59; 16.86–19.92), and negative in the MV group (− 5.32; − 15.16- − 0.77; p = 0.0022) (Fig. 3). The microglia percentage, combining both pro-inflammatory and anti-inflammatory cells, was lower in the NV group (10.12; 8.93–10.65) compared to the MV group (36.17; 30.71–48.27; p = 0.0022) (Fig. 4A–C). A significantly higher percentage of microglia cells with anti-inflammatory characteristics was observed in the NV group (84.40; 80.49–86.27) than the MV group (80.63; 71.59–81.93, p = 0.0411) (Fig. 4D). The percentage of microglia cells with pro-inflammatory characteristics was higher, tending towards significance, in the MV group (18.63;17.37–27.38) than in the NV group (15.60;13.73–19.51, p = 0.0649) (Fig. 4E).

**Figure 5:**
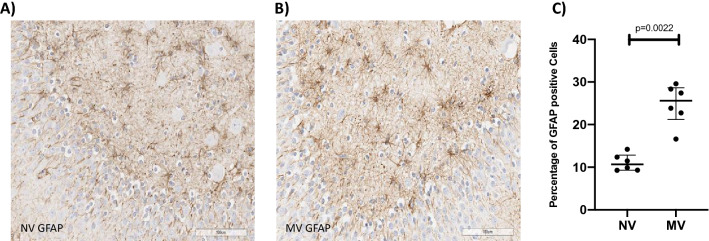
Slide of an NV subject’s hippocampus (**A**) and an MV subject’s hippocampus (**B**) after staining by GFAP, showing normal cells (blue) and GFAP-positive cells (brown). (**C**) Dot plot showing percentages of GFAP-positive cells (astrocytes), 10.69 (9.31–12.85) in the NV group and 25.53 (21.21–28.66) in the MV group, p = 0.0022 (n = 6 per group).

The percentage of GFAP-positive astrocytes, as a proportion of all reactive astrocytes, was lower in the NV group (10.69; 9.31–12.85) compared to the MV group (25.63; 21.21–28.66; p = 0.0022) (Fig. 5A–C). The percentage of cells stained positive by IL-1α antibody demonstrated a significant difference between the NV group (0.00; 0.00–0.00) and the MV group (40.73; 34.66–49.43; p = 0.0048). The percentages of cells stained by IL-6 antibody and TNF-α antibody demonstrated no significant differences between groups.

### Serum markers

**Figure 6:**
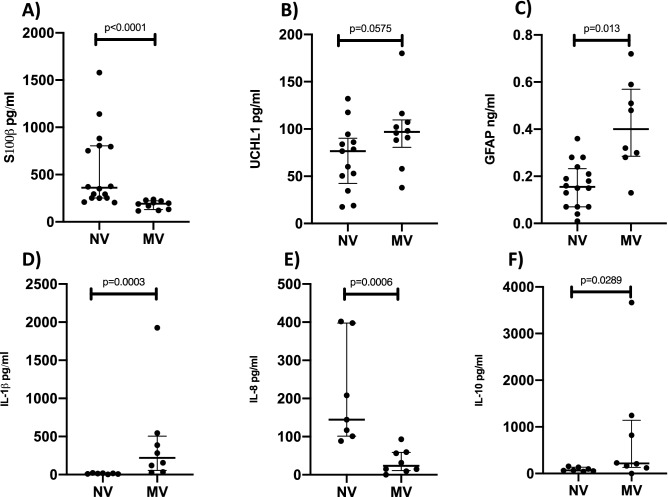
(**A**) Dot plot showing serum concentrations of S100β, 360.90 pg/ml (252.10–803.60) in the NV group and 193.10 pg/ml (129.20–223.30) in the MV group, p < 0.0001 (n = 10–16 per group). (**B**) Dot plot showing serum concentrations of UCHL1, 76.57 pg/ml (42.48–90.26) in the NV group and 96.96 ng/ml (80.65–109.60) in the MV group, p = 0.0575 (n = 10–13 per group). (**C**) Dot plot showing serum concentrations of GFAP, 0.15 ng/ml (0.07–0.23) in the NV group and 0.40 ng/ml (0.28–0.57) in the MV group, p = 0.0013 (n = 8–16 per group). (**D**) Dot plot showing serum concentrations of IL-1β, 13.39 pg/ml (7.72–17.68) in the NV group and 219.60 pg/ml (56.23–505.40) in the MV group, p = 0.0003 (n = 7–8 per group). (**E**) Dot plot showing serum concentrations of IL-8, 144.50 pg/ml (100.90–397.90) in the NV group and 23.40 pg/ml (10.88–58.51) in the MV group, p = 0.0006 (n = 7–8 per group). (**F**) Dot plot showing serum concentrations of IL-10, 67.92 pg/ml (55.60–134.80) in the NV group and 217.50 pg/ml (131.30–1,140.00) in the MV group, p = 0.0289 (n = 7–8 per group).

S100β serum concentration was significantly higher in the NV group (360.90 pg/ml; 252.10–803.60) than in the MV group (193.00 pg/ml; 129.20–223.30; p < 0.0001) (Fig. 6A). UCHL1 serum concentration showed a trend towards being lower in the NV group (76.57 pg/ml; 42.48–90.26) than in the MV group; (96.96 pg/ml; 80.65–109.60; p = 0.0575) (Fig. 6B). GFAP serum concentration was significantly lower in the NV group (0.15 ng/ml; 0.07–0.23) than in the MV group (0.40 ng/ml; 0.28–0.57; p = 0.0013) (Fig. 6C). IL-1β serum concentration was significantly lower in the NV group (13.39 pg/ml; 7.72–17.68) than in the MV group (219.60 pg/ml; 56.23–505.40; p = 0.0003) (Fig. 6D). IL-8 serum concentration was significantly higher in the NV group (144.50 pg/ml; 100.90–397.90) than in the MV group (23.40 pg/ml; 10.88–58.51; p = 0.0006) (Fig. 6E). IL-10 serum concentration was significantly lower in the NV group (67.92 pg/ml; 55.60–134.80) than in the MV group (217.50 pg/ml; 131.30–1,140.00; p = 0.0289) (Fig. 6F). Serum analyses of NSE, TNF-α, IL-1α and IL-6 demonstrated no significant differences between groups.

### PaO_2_/FiO_2_ ratio

**Figure 7:**
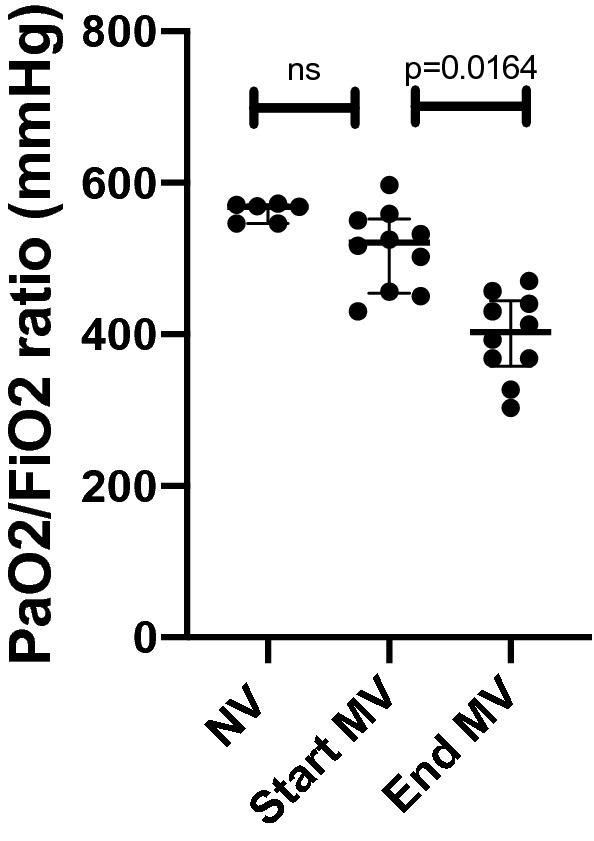
Dot plot showing the PaO_2_/FiO_2_ ratio of the NV group (568 mmHg; 546–571), the PaO_2_/FiO_2_ ratio of the MV group at the start of the experiment (521 mmHg; 454–552) and the PaO_2_/FiO_2_ ratio of MV group at the end of the experiment (403 mmHg; 357–444). No statistically significant difference was found when comparing the PaO_2_/FiO_2_ ratio of the NV group (568 mmHg; 546–571) to the PaO_2_/FiO_2_ ratio of the MV group at the start of the experiment (521 mmHg; 454–552, p = 0.3700). The PaO_2_/FiO_2_ ratio of MV group at the end of the experiment (403 mmHg; 357–444) when compared to the PaO_2_/FiO_2_ ratio of the MV group at the start of the experiment (521 mmHg; 454–552, p = 0.0164) was statistically significant.

 No statistically significant difference was found when comparing the PaO_2_/FiO_2_ ratio of the NV group (568 mmHg; 546–571) to the PaO_2_/FiO_2_ ratio of the MV group at the start of the experiment (521 mmHg; 454–552, p = 0.3700). The PaO_2_/FiO_2_ ratio of the MV group at the end of the experiment (403 mmHg; 357–444 mmHg) was statistically different from the PaO_2_/FiO_2_ ratio of the MV group at the start of the experiment (521 mmHg; 454–552, p = 0.0164) (Fig. 7).

### Lung injury score

**Figure 8:**
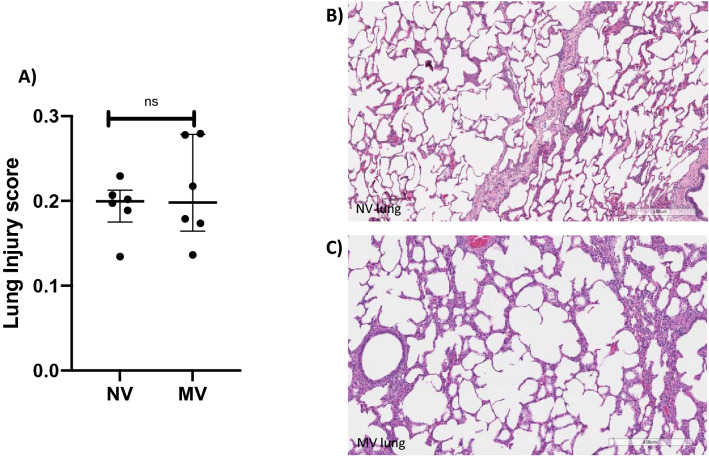
(**A**) Dot plot showing the lung injury scores of the NV group (0.19; 0.17–0.21) and the lung injury scores of the MV group (0.19; 0.16–0.27) with no statistical difference between the groups, p = 0.8182. Lung histology slides stained by hematoxylin and eosin, NV subject (**B**) and MV subject (**C**).

No statistically significant difference was found when comparing the lung injury score of the NV group (0.19; 0.17–0.21) to the lung injury score of the MV group (0.19; 0.16–0.27, p = 0.8182) (Fig. 8).

## Discussion

Our results show that lung-protective MV with sedation and immobility in the supine position is associated with neurological injury and neuroinflammation. This is a concerning finding, as despite our best efforts to use “gentle” ventilation and judicious sedation, our pre-clinical work demonstrates that we are likely injuring the brain.

Greater hippocampal inflammation and apoptosis could be clearly observed, both histologically and biochemically, after 50 h of lung-protective MV and sedation. This is important, as hippocampal inflammation and apoptosis have been shown to be present in cognitively impaired subjects, both in clinical and preclinical studies^7,11^. Van Munster et al. showed a higher presence of microglia and astrocytes in the hippocampus of deceased patients with delirium than in those of deceased patients without delirium during hospitalization^7^. In a preclinical study, Chen et al. demonstrated that six hours of MV in mice was associated with lower cognitive scores than in a non-ventilated comparator group, and the mice with lower cognitive scores had greater levels of hippocampal neuroinflammation and cellular apoptosis^11^. Our results do not show the extent to which this injury manifests in the clinical realm. The injury level observed was analogous to that reported previously in studies that investigated the association between impaired cognition and histological hippocampal changes. Our data may provide insights into the biological foundation of neurological insult and cognitive dysfunction after critical illness^7,11^.

The higher mitotic index in the MV group (26.04 in the MV group vs. 19.07 in the NV group) may be the result of a protective mechanism against tissue injury during MV and sedation. Neurogenesis has been reported to be higher during physical activity, with epilepsy and after ketamine administration, and lower when being sedentary, and during inflammation, midazolam administration and propofol administration^3,8,12,13^. In the present study we are not able to isolate the impact of each variable on the neurogenesis rate. However, the specific contribution of each variable may ultimately not be relevant, as these three factors (MV, deep sedation and immobilization in the supine position) coexist in deeply sedated patients in the ICU who, by necessity, require MV and are also consequently immobilized. Although neurogenesis is essential to maintaining neural health, the mitotic index alone tells an incomplete story because tissue homeostasis is achieved by balancing newborn cells and cellular death.

Cellular apoptosis is a natural process that occurs as part of a cycle of programmed cell death and renewal throughout the body^5^. This process can be potentiated by external signals, leading to cellular tissue dysregulation^5^. In rodents, higher hippocampal apoptosis has been shown after less than four hours of injurious high-tidal-volume MV when compared to non-ventilated animals^5^. Greater hippocampal apoptosis was also demonstrated in mechanically ventilated subjects in the Trendelenburg position than mechanically ventilated subjects in the supine position^14^. Moreover, propofol and ketamine were also reported to induce neurotoxicity in a rodent model, where the use of these drugs resulted in neuronal apoptosis^3,12^. Conversely, midazolam seems to protect brain cells by reducing reactive oxidative stress and by supressing c‑Jun N‑terminal kinases, an important protein in both intrinsic and extrinsic apoptotic pathways^15^. Our data showed a higher level of apoptosis in the MV group than in never-ventilated pigs (apoptotic indices of 31.70 vs. 0.96 in the MV and NV groups, respectively) which could reasonably be attributed to the effects of MV, sedation or immobility in the supine position either in isolation or in synergy. The negligible level of apoptosis in the NV group (0.96) is likely part of the natural cellular renewal process in the body, described in the literature^5^. The apoptotic index alone has been identified as an important marker of acquired brain injury, however combining it with the neurogenesis data might provide an overall perspective of the cellular tissue balance.

Brain tissue must maintain a balance between the number of new cells generated and those that die. The turnover index is a measure of tissue cellular balance between cell gain (mitotic index) and loss (apoptotic index)^9^. This balance is vital for healthy neurological function, being clinically relevant as recent studies have shown a positive correlation between the presence of hippocampal atrophy, mechanical ventilation and mood disorders in ARDS patients^16,17^. Our data showed more cell death (apoptotic indices of 31.70 vs. 0.96) and significantly less net cell replacement (turnover indices of − 5.32 vs. + 18.59) in the MV group than in the NV group. The comparatively large positive turnover index in the NV group is likely a consequence of the subjects being juveniles^8^. The negative turnover index in the MV group is a concerning finding, since all subjects at this stage of development would be expected to have a pronounced resilience against tissue insult^8^. It may be argued that if this were to continue over an extended period it would have led to a net loss of neural cells. Therefore the variables studied here might have affected the cellular balance in the MV group, with higher mitosis levels being inadequate to compensate for the greater apoptosis levels observed in this group. It is likely that the magnitude of this injury is time-dependent, but it is not clear to what extent the injury is also affected by the characteristics of each variable. Although turnover index was historically used to describe neoplastic tissue changes, we believe that it gives a more comprehensive understanding of the dynamic cellular process happening in the hippocampus during MV than apoptotic index alone^9,18^. Taken in isolation, the higher mitosis rate seen in the MV group may be interpreted as beneficial; however, when viewed in the context of the higher rates of apoptosis, it becomes apparent that it is a reaction to injury after 50 h of MV and sedation, and is likely not fully adequate to maintain overall cellular balance. As the turnover index has not been previously validated in studies of brain injury, its inclusion here is meant to reflect the balance between cell death and regeneration in a single number for ease of comparison.

The injury observed might also impact other cell populations that play a critical role in maintaining a healthy neural environment, including microglia and astrocytes^7,19^. Greater numbers of microglia and astrocytes have been correlated with hippocampal dysfunction^7^. For example, brain autopsies from ARDS patients with acute cognitive impairment have shown elevated numbers of activated microglia and reactive astrocytes in the hippocampus^7,20^. Our data showed that the relative proportion of microglia in the hippocampus, compared to the overall cell population was greater in the MV group (36.12%) than the NV group (10.12%; p = 0.0022). These cells can secrete IL-1α, an inflammatory protein that can also stimulate neurogenesis and is associated with apoptosis^21^. We found greater IL-1α levels in the MV group (40.73%) than in the NV group (0.00%; p = 0.0048). The proportion of reactive astrocytes was also greater in the MV group (25.63%) than in the NV group (10.69%; p = 0.0022). The hippocampus of animals undergoing MV and sedation demonstrated a recruitment and activation of microglia as well as astrocytes that likely reflected an initial brain insult over the course of the experiment and also contributed to its propagation.

Serum analysis of small proteins primarily expressed by astrocytes and by neurons have been used to show either blood–brain barrier integrity or neuronal injury^22^. In the MV group, we found significantly greater serum concentrations of GFAP, the most specific protein to identify brain injury, in the MV group (0.40 ng/ml) in comparison to the NV group (0.15 ng/ml; p = 0.0013)^22,23^. Similarly, the levels of UCHL1 were also greater in the MV group (96.96 pg/ml) than in the NV group (76.57 pg/ml; p = 0.0575). The greater levels of these two proteins indicate the considerable level of neuronal damage in the MV group, which was also observed in the histological analysis. Conversely, S100β levels, a calcium binder protein that might be used to evaluate blood–brain barrier integrity, were higher in the NV group than in the MV group (193.10 pg/ml vs. 360.90 pg/ml). Higher concentrations of this protein in the NV group might be an effect of sedation leading to immobility, since S100β is also affected by muscle activity^24–26^. Studies have shown that after physical activity S100β levels increase dramatically, supporting the hypothesis that immobility after sedation might have an effect on S100β levels^26,27^. Thus, it may be concluded that neuronal insult occurred due to a combination of lung-protective MV, sedation and physical immobility.

The mechanism that triggered the observed insult is unknown. However, systemic inflammation from either MV-induced biotrauma or immobility might have played a role. Serum concentration of IL-1β, an important pro-inflammatory cytokine, was found to be significantly greater in the MV group (219.60 pg/ml) than in the NV group (13.39 pg/ml). Serum concentration of IL-10, an anti-inflammatory protein, showed considerably greater serum concentration in the MV group (217.50 pg/ml) than in the NV group (67.92 pg/ml), which likely represents a protective mechanism. The level of IL-8 was also significantly higher in the NV group (144.50 pg/ml) than in the MV group (23.40 pg/ml). This may have been a consequence of both midazolam and propofol, as the release of IL-8 has been shown to be supressed by these drugs, which were only administered to the MV subjects^28–30^. It is unclear to what extent this cytokine profile either represents a direct injury to the brain mediated by inflammation, or is solely a result of lung inflammation^5,19^.

Our findings have reasonable biological plausibility and the data consistently reflects a statistically significant injury associated with lung-protective MV, sedation and immobility in the supine position. Although preclinical studies can control for many factors, our study has some important limitations. Firstly, our model used animals with normal lungs and no substantial lung injury was observed during the study. Specifically, lung injury scores were similar between the groups and PaO_2_/FiO_2_ ratios were above 400 mmHg. Many ICU patients have either some degree of lung injury or other systemic inflammatory processes; however, by isolating healthy subjects it allowed us to control for potential confounding factors that may occur during inflammatory states, and thereby isolate the variables of interest studied here. It is reasonable to anticipate that inflammatory processes unrelated to MV, sedation and immobility would likely potentiate the injury observed in this study, and remain to be studied. Due to the intense nature of the experiment, relatively low numbers of subjects were studied, which may have limited our findings. Additionally, our study did not look at hippocampal connectivity and the maturity of synaptic connections. Another limitation was the use of the turnover index, which currently is not a well-established measure of cellular balance in cancer-free tissue; it may be argued that this index does not exclusively measure neuronal cellular balance in our model. However, this index may be considered useful to reflect the impact on the brain of the variables studied here, since this index was analyzed in both groups, and the sampled areas used for the calculation of this index were densely neuronally populated, thereby reducing potentially false-positive apoptosis results. Moreover, there is a possibility that our experimental protocol itself led to the results we observed, independent of MV and sedation. However, this is unlikely, as our findings are supported by other research into MV and sedation^5,12^. Additionally, although our team confirmed that asynchrony was not present during the experiment, reverse triggering as a possible source of injury during the experiment cannot be ruled out. While patient self-induced lung injury has been recently reported in the literature, no clinical signs were observed in the mechanically ventilated subjects during the experiment. Finally, the clinical relevance of these findings still requires further investigation.

## Conclusion

In our preclinical model, 50 h of lung-protective MV, sedation and immobility in the supine position were associated with neurological injury, characterized by a net loss of hippocampal cells. Elevated levels of apoptosis overwhelmed the compensatory higher level of neurogenesis, along with greater hippocampal inflammation as well as greater levels of markers in the serum for neuronal damage and astrocyte damage. This is important, as this injury was similar in magnitude to that observed preclinically and clinically in cognitively impaired subjects in other studies. This data provides new insights into the effects of MV, sedation and immobility on the brain, which need to be further explored in both preclinical and translational studies. Future preclinical studies could extend this work into a more relevant lung-injured model to explore whether lung injury in association with the variables studied here have a synergistic effect on the observed insult to the brain.

## Methods

### Animals

Juvenile Yorkshire pigs (4–5 months old) were procured, housed, maintained, and studied in accordance with the local Animal Care Committee guidelines after UBC Ethics Committee and Animal Care Committee approvals.

### Experimental protocol

#### NV group

Subjects were studied in pairs, and each animal was monitored during the experiment, utilizing an ICU monitor for heart rate, oximetry and EKG tracings^31^. All animals were kept in the supine position with head elevation of zero degrees.

Ketamine was given once in a bolus (20 mg/kg) to induce conscious sedation, meaning sedation without inducing apnea and without the need for intubation. Supplemental oxygen was supplied via facial mask at 2–4 l/min to maintain oxygen saturation of 92% or greater. Vitals were monitored to ensure hemodynamic stability at all times prior to the euthanasia process.

Once adequate sedation had been established, a central line catheter was inserted in the subclavian vein to assist exsanguination post-euthanasia. The time course between the start of the procedure and euthanasia was around 30 min.

Six pigs had tissue harvested, making up the never-ventilated group (NV group) reported on here for histological analysis results. Ten additional NV animals, each of which was handled following the same experimental protocol, were available for blood sampling as part of other work, and we elected to include all available samples in our analysis, totaling up to 16 serum samples analyzed in the NV group.

#### MV group

Subjects were also studied in pairs, and each animal was monitored during the experiment, utilizing an ICU monitor for heart rate, oximetry, capnometry and EKG tracings^31^. All animals were kept in the supine position with head elevation of zero degrees.

Ketamine was given in bolus (20 mg/kg) to induce conscious sedation, meaning sedation without inducing apnea. Supplemental oxygen was supplied via facial mask at 2–4 l/min to maintain oxygen saturation of 92% or greater. Subsequently, the pigs were orally intubated.

Ten oral-endotracheally intubated pigs were ventilated with lung-protective volume control settings (8 ml/kg tidal volume), receiving sedative drugs commonly used in ICUs (MV group); protocol previously described by our group^31^. These ten mechanically ventilated pigs had blood samples taken at the end of the experiment and had tissue harvested for histological analysis. We report here all available results from our analysis of samples from the MV group.

Vitals were monitored to ensure hemodynamic stability at all times prior to the euthanasia process. Once adequate sedation had been established, a central line catheter was inserted in the subclavian vein. The central line was used to deliver fluid during the experiment, to measure the central venous pressure, and to assist exsanguination post-euthanasia. An arterial line also was placed in the femoral artery, in all subjects in the MV group, to continuously measure arterial pressure and facilitate blood gas sampling. Additionally, in the MV group, blood gases were taken every six hours to analyze PaO_2_, PaCO_2_, HCO_3_ and pH (see online supplement Table 2). Slight alkalosis (pH 7.40–7.45) was favored to reduce breathing drive and was achieved through slight alterations in minute ventilation. End tidal CO_2_ was monitored in all MV subjects (see online supplement Table 2).

Ventilation data was recorded and visually reviewed breath-by-breath for spontaneous breathing, single-breath episodes, reverse triggering and ventilation asynchrony post-experiment. Sedation was assessed by bispectral index and by clinical signals of sedation^32^.

All subjects had bladder catheterization to quantify urine output during the course of the study. Fluid was delivered targeting positive total fluid balance in a range of 0.1–2.0 ml/kg/day. The time between the initiation of MV and euthanasia was 50 h. Brains were harvested following our previously published methodology^33^.

### Ventilation settings

For the MV group, ventilators used were standard adult ICU models, Dräger Evita XL and Puritan Bennett 840. Our study protocol followed the ARDSnet guideline for lung-protective ventilation: tidal volume in the range of 6–8 ml/kg and plateau pressure less than 30 cmH_2_O^34^. Due to dead-space considerations, the subjects were ventilated at 8 ml/kg. Additionally, ventilation parameters in volume control mode were set to a PEEP of 5 cmH_2_O, respiratory rate of 20 breaths/minute (18–22), triggering 3 l/min, sensitivity P/V of 3, I:E ratio of 1:3 (1:2.5–1:3.5) and an FiO_2_ of 30%. Slight alkalosis was favored (pH 7.40–7.45) to ablate respiratory drive. All MV subjects had blood gas parameters in the normal range during the 50 h of the experiment (see supplement Table 2).

### Deep sedation

All subjects in the MV group had sedation checked hourly by clinical parameters (i.e. jaw tonus, quadriceps tonus, heart rate variation and blood pressure variation) and by Bi-Spectral Index (BIS) assessment. Bi-Spectral Index measures bispectral EEG, which is used regularly in humans, and is validated in pigs for assessment of sedation levels^32,35^. BIS scores of 65 or less were considered deep sedation in our study, and when BIS was greater than 65, sedation was increased to achieve the desired sedation level. Sedation was achieved by continuous infusion of propofol (100–150 mg/kg/hour), combined with continuous infusion of a solution containing midazolam (0.1–0.5 mg/kg) plus fentanyl (30–100 μg/kg/hour). Bolus of ketamine (11–33 mg/kg/dose) was used when appropriate (see supplement Table 3).

### Hippocampal sampling and preparation

Molecular, granular, and sub-granular layers were randomly sampled in the dentate gyrus, and the pyramidal layer was randomly sampled in the CA1 and CA3 hippocampal areas (all in the same slide section). All the sampling areas came from the same slide section for each marker. Equal-sized sample areas (200 µm by 200 µm) and equal numbers of areas (21 per subject per marker) were analyzed in all hippocampal samples for all subjects. An independent laboratory (Wax-it Histology Services Inc.), blinded to sample group, performed immunochemistry preparation and processing.

### Hippocampal immunohistochemistry

The mitotic and apoptotic indexes were determined by calculating the percentages of doublecortin- and TUNEL-positive cells, respectively. Total number of positive-stained cells was divided by the total cell population per marker. Turnover index was calculated by subtracting apoptotic index value from mitotic index value.

Microglia percentage was defined as the total number of IBA-1-positive cells divided by the total cell population (IBA-1-positive cells plus IBA-1-negative cells) per slide. Microglia cells were divided into two populations based on biometric cellular characteristics^36^. Anti-inflammatory microglia cells were defined as having roundness between 0.5–1.0 and soma size 30–60 µm^2^, and pro-inflammatory microglia cells were defined as having roundness between 0.0–0.5 and soma size^36^ 60–90 µm^2^. To further evaluate inflammation, GFAP, TNF-α, IL-1α and IL-6, were analyzed in the hippocampus.

### Cell counting and cell classification

Cells were counted by machine-learning software (ImageJ) (see supplement Figure A). To mitigate bias, our study used an automatic and replicable method to count and classify cells previously established in the literature (see supplement Figure B)^37^. A team member, blinded to study group, also verified 10% of our results following the same cell-counting protocol to reinsure replicability and data integrity. Both investigators independently trained the machine-learning software following the same protocol. In addition, the areas sampled were the same for both examiners, and the only difference was that the second investigator was blinded to study group.

### Serum samples

Both groups had samples taken to analyze serum concentration of S100β, Neuron Specific Enolase (NSE), GFAP, Ubiquitin C-terminal Hydrolase L1 (UCHL1), TNF-α, IL-1α, IL-1β, IL-6, IL-8 and IL-10. An independent laboratory, Eve Technologies Corporation, blinded to study group, processed the samples.

### PaO_2_/FiO_2_ ratio

Both groups had arterial blood gas samples taken, allowing the PaO_2_/FiO_2_ ratio to be calculated. The PaO_2_/FiO_2_ ratio was calculated by dividing the blood oxygen partial pressure (PaO_2_) by the fraction of inspired oxygen (FiO_2_). The NV group had arterial blood samples taken once, and the MV group had arterial blood samples taken every six hours to ensure proper blood oxygenation and to check the acid–base homeostasis. The first arterial blood samples taken from the MV group after the beginning of the experiment were categorized as “start of the experiment” and the last arterial blood samples taken from the MV group before the end of the experiment were categorized as “end of the experiment” for the purposes of calculating the PaO_2_/FiO_2_ ratio.

### Lung histology

Six NV subjects and six MV subjects had their left lungs harvested. Each lung had five standardized areas sampled (see supplement Diagram 1). An independent laboratory (Wax-it Histology Services Inc.), blinded to sample group, stained the slides with haematoxylin and eosin. Blinded samples were scored for lung injury according to the method adapted from Matute-Bello^38^.

### Statistical analysis

Statistical analyses were non-parametric, utilizing the Mann–Whitney U test or paired Wilcoxon signed-rank test when appropriate, using GraphPad Prism 8.4.2 software. Prevalence of each marker was measured for each subject; for serum concentrations, duplicate samples from each subject were averaged. Data are expressed as median and interquartile range (IQR). P-values ≤ 0.05 were considered statistically significant.

A power calculation was performed using α = 0.01 and β = 0.90, based on data from González-López, et al. (2013)^5^ indicating two subjects per arm would be necessary to achieve statistical power. As this would pose risk of bias, the number of samples was increased to at least six per arm.

### Ethics approval and consent to participate

The study was conducted in accordance with Canadian Council for Animal Care guidelines and approved by the University of British Columbia Animal Ethics Committee.

## Supplementary Information

Supplementary information.

## Data Availability

The datasets used and/or analysed during the current study are available from the corresponding author on reasonable request.
