# Supplementary material for: Brain injury after 50 h of lung-protective mechanical ventilation in a preclinical model
Source: Sci Rep. 2021 Mar 3;11:5105. doi: 10.1038/s41598-021-84440-1 (PMC7930247; doi:10.1038/s41598-021-84440-1)
Supplement: Supplementary file 1 — Supplementary information. [file 41598_2021_84440_MOESM1_ESM.docx]

**Title: Brain injury after 50 hours of lung-protective mechanical ventilation in a preclinical model**

Thiago G. Bassi^1,2^, Elizabeth C. Rohrs^1,3^, Karl C. Fernandez^1,3^, Marlena Ornowska^1^, Michelle Nicholas^1,3^, Matt Gani^2^, Doug Evans^2^, Steven C. Reynolds^1,3^

^1^ Simon Fraser University, ^2^ Lungpacer Medical, Inc., ^3^ Fraser Health Authority, Royal Columbian Hospital

Corresponding author: Dr. Steven Reynolds [sreynolds.md@gmail.com](about:blank)

Site Medical Director, Royal Columbian Hospital

260 Sherbrooke Street, New Westminster, B.C. Canada. V3L 3M2

**Pre-experiment procedures:**

A health check-up was completed one week prior to the experiments, which included clinical examination, blood work and baseline EKG for both groups. Animals were housed in pairs according to best practice guidelines.

Animal weights prior to the start of the experiment are shown in Table 1.

| NV Group (all female) | | MV Group (all female) | |
| --- | --- | --- | --- |
| Subject | Weight (kg) | Subject | Weight (kg) |
| 1 | 50 | 1 | 43 |
| 2 | 53 | 2 | 55 |
| 3 | 56 | 3 | 47 |
| 4 | 55 | 4 | 56 |
| 5 | 56 | 5 | 63 |
| 6 | 53 | 6 | 64 |
| --- | --- | 7 | 57 |
| --- | --- | 8 | 57 |
| --- | --- | 9 | 66 |
| --- | --- | 10 | 66 |
| Median | 54 | Median | 57 |
| --- | p=0.1104 | | |

Table 1. Subject weight. No statistically significant difference was found between the groups, p=0.1104.

**Initial sedation and catheterization, and animal monitoring during the experiment:**

1. NV group

Subjects were studied in pairs, and each animal was monitored during the experiment, utilizing an ICU monitor for heart rate, oximetry and EKG tracings.^1^ All animals were kept in the supine position with head elevation of zero degrees.

Ketamine was given once in a bolus (20 mg/kg) to induce conscious sedation, meaning sedation without inducing apnea and without the need for intubation. Supplemental oxygen was supplied via facial mask at 2-4 l/min to maintain oxygen saturation of 92% or greater. Vitals were monitored to ensure hemodynamic stability at all times prior to the euthanasia process.

Once adequate sedation had been established, a central line catheter was inserted in the subclavian vein to assist exsanguination post-euthanasia. The time course between the start of the procedure and euthanasia was around 30 minutes.

Six pigs had tissue harvested, making up the never-ventilated group (NV group) reported on here for histological analysis results. Ten additional NV animals, each of which was handled following the same experimental protocol, were available for blood sampling as part of other work, and we elected to include all available samples in our analysis, totaling up to 16 serum samples analyzed in the NV group.

1. MV group

Subjects were also studied in pairs, and each animal was monitored during the experiment, utilizing an ICU monitor for heart rate, oximetry, capnometry and EKG tracings.^1^ All animals were kept in the supine position with head elevation of zero degrees.

Ketamine was given in bolus (20 mg/kg) to induce conscious sedation, meaning sedation without inducing apnea. Supplemental oxygen was supplied via facial mask at 2-4 l/min to maintain oxygen saturation of 92% or greater. Subsequently, the pigs were orally intubated.

Ten oral-endotracheally intubated pigs were ventilated with lung-protective volume control settings (8 ml/kg tidal volume), receiving sedative drugs commonly used in ICUs (MV group); protocol previously described by our group.^1^ These ten mechanically ventilated pigs had blood samples taken at the end of the experiment and had tissue harvested for histological analysis. We report here all available results from our analysis of samples from the MV group.

Vitals were monitored to ensure hemodynamic stability at all times prior to the euthanasia process. Once adequate sedation had been established, a central line catheter was inserted in the subclavian vein. The central line was used to deliver fluid during the experiment, to measure the central venous pressure, and to assist exsanguination post-euthanasia. An arterial line also was placed in the femoral artery, in all subjects in the MV group, to continuously measure arterial pressure and facilitate blood gas sampling. Additionally, in the MV group, blood gases were taken every six hours to analyze PaO_2_, PaCO_2_, HCO_3_ and pH (Table 2). Slight alkalosis (pH 7.40-7.45) was favored to reduce breathing drive and was achieved through slight alterations in minute ventilation. Also, end tidal CO_2_ was monitored in all MV subjects (Table 2).

Ventilation data was recorded and checked breath-by-breath for spontaneous breathing, single-breath episodes, reverse triggering and ventilation asynchrony post-experiment. Sedation was assessed by bispectral index and by clinical signals of sedation.^2^

All subjects had bladder catheterization to quantify urine output during the course of the study. Fluid was delivered targeting positive total fluid balance in a range of 0.1-2.0 ml/kg/day (1.1 ml/kg/day; 0.59-1.58). The time between the initiation of MV and euthanasia was 50 hours. Brains were harvested following our previously published methodology.^3^

Table 2. Blood gas results for the MV group.

|  | **pH** | **PaCO_2_ (mmHg)** | **PaO_2_ (mmHg)** | **HCO3** (**mmol/L)** | **etCO_2_ (%)** |
| --- | --- | --- | --- | --- | --- |
| MV group  Median (IQR) | 7.43  (7.39-7.45) | 44.7  (42.0-47.6) | 136.0  (123.0-147.0) | 31.0  (29.2-31.4) | 5.5  (5.1-5.7) |

**Ventilation settings:**

For the MV group, ventilators used were standard adult ICU models, Dräger Evita XL and Puritan Bennett 840. Our study protocol followed the ARDSnet guideline for lung-protective ventilation: tidal volume in the range of 6-8 ml/kg and plateau pressure less than 30 cmH_2_O.^4^ Due to dead-space considerations, the subjects were ventilated at 8 ml/kg. Additionally, ventilation parameters in volume control mode were set to a PEEP of 5 cmH_2_O, respiratory rate of 20 (18-22), triggering 3l/min, sensitivity P/V of 3, I:E ratio of 1:3 (1:2.5-1:3.5) and an FiO_2_ of 30%. Slight alkalosis was favored (pH 7.40-7.45) to ablate respiratory drive. All MV subjects had blood gas parameters in the normal range during the 50 hours of the experiment.

**Deep sedation:**

All subjects in the MV group had sedation checked hourly by clinical parameters (i.e. jaw tonus, quadriceps tonus, heart rate variation and blood pressure variation) and by Bi-Spectral Index (BIS) assessment. Bi-Spectral Index measures bispectral EEG, which is used regularly in humans, and is validated in pigs for assessment of sedation levels.^2,5^ BIS scores of 65 or less were considered deep sedation in our study, and when BIS was greater than 65, sedation was increased to achieve the desired sedation level. Sedation was achieved by continuous infusion of propofol (100-150 mg/kg/hour), combined with continuous infusion of a solution containing midazolam (0.1-0.5 mg/kg) plus fentanyl (30-100 μg/kg/hour). Bolus of ketamine (11-33 mg/kg/dose) was used when appropriate. Total amounts of all sedative drugs administered are shown, per MV subject, in Table 3.

| MV Subject | Weight (kg) | Total amounts administered | | | |
| --- | --- | --- | --- | --- | --- |
|  |  | Propofol  (mg) | Fentanyl  (μg) | Midazolam  (mg) | Ketamine  (mg) |
| 1 | 43 | 116.40 | 400.00 | 40.00 | 240.00 |
| 2 | 55 | 104.94 | 1026.80 | 102.68 | 785.70 |
| 3 | 47 | 72.82 | 446.45 | 44.65 | 1,292.59 |
| 4 | 56 | 59.69 | 803.55 | 80.36 | 1,246.43 |
| 5 | 63 | 114.01 | 491.05 | 49.11 | 1,230.00 |
| 6 | 64 | 135.67 | 803.50 | 80.35 | 1,614.29 |
| 7 | 57 | 112.55 | 575.05 | 57.51 | 3,000.00 |
| 8 | 57 | 126.06 | 471.45 | 47.15 | 4,700.00 |
| 9 | 66 | 68.7 | 372.14 | 55.8 | 1,400.00 |
| 10 | 66 | 52.2 | 240.00 | 36.0 | 880.00 |
| Median | 57 | 108.745 | 481.25 | 52.455 | 1,269.51 |

Table 3. Sedative drugs administered for each MV subject during the experiment.

**Euthanasia:**

At the end of the experiment, with the aid of an animal health technician, the animals were euthanized using a bolus of ethanol followed by exsanguination.

**Brain harvesting:**

At necropsy, brain samples were obtained following our methodology previously published in the literature.^3^ Brains were harvested after a hexagonal craniotomy followed by proper dissection of the surgical planes. The brainstem and cerebellum were resected and then the encephalus was harvested following the methodology previously published by our group.^3^ The left ventral hippocampus was dissected and excised for analysis. The right brain hemisphere was resected and stored in its entirety. These samples were preserved and further processed by an external laboratory (Wax-it Histological Services Inc.), which was blinded to study group for the samples, for later analysis. All brain samples from the NV group were stored in formalin. Samples from four brains from the MV group were stored frozen, and samples from the other six brains from the MV group were stored in formalin.

**Immunohistochemistry:**

An independent laboratory, Wax-it Histology Services Inc., which was blinded to study group for the samples, processed the hippocampal slides using methodology previously described in the literature.^6^ The antibodies used were: TUNEL – Millipore S7100, Doublecortin – Abcam ab18723, IBA-1 – Wako019-19741, GFAP – Dako Z0334, IL-1α – R&D Systems AF-400-NA, IL-6 – Abcam ab 6672, and TNF-α – Abcam ab6671. The general protocol entailed incubation of slides in a 60^o^C oven for 60 minutes, or until paraffin from the slide had melted. This was followed by washes of xylene, alcohol and TBS-plain. The next steps were an endogenous block of peroxidase, protein block and incubation of the primary antibody at 4^o^C. A second antibody was used if necessary, depending on the targeted protein to be analyzed. Finally, the slides were rinsed with water to stop DAB (3,3′-Diaminobenzidine) reaction until the DAB became clear.

**Hippocampal regions sampled:**

Molecular, granular, and sub-granular layers were randomly sampled in the dentate gyrus, and the pyramidal layer was randomly sampled in the CA1 and CA3 hippocampal areas (all in the same slide section). The number of areas sampled (21) and the cross-sectional areas (200 μm by 200 μm) were identical between subjects and between groups.

**ImageJ cell counting:**

An ImageJ plug-in, Trainable Weka Segmentation, was used to count and classify cells. A unique software classifier was used to count and to classify cells for each hippocampal immunohistochemistry marker.^7,8^ For example, TUNEL had a different software classifier than did doublecortin. In each slide, the machine-learning software differentiated cells stained positive by the immunohistochemistry marker used from non-positive cells (normal cells) (Figure A).


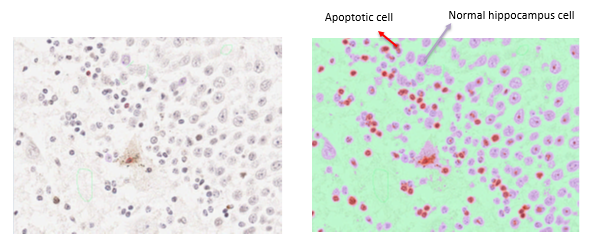


Figure A. Slide of an MV subject’s hippocampus without the ImageJ filter (left), showing normal cells (blue) and TUNEL-positive cells (brown); the same slide of an MV subject’s hippocampus with ImageJ filter (right), showing how the software classified normal cells (magenta) and TUNEL-positive cells (red).

An investigator, who was blinded to study group for the samples, doubled-checked approximately 200,000 cells to ensure data integrity and replicability. Both investigators trained the machine learning software following the same protocol. In addition, the areas sampled were the same for both examiners, and the only difference was that the second investigator was blinded to study group. Statistical analysis, comparing the data collected from the two different examiners (blinded vs. non-blinded) revealed that the data were significantly paired, p<0.0001, and that the data collected were also similar, p=0.1190 (Figure B). Analysis used the paired Wilcoxon signed-rank (non-parametric) test, with GraphPad Prism 8.4.2 software.

Figure B. Dot plot showing the distribution of data collected from two different examiners, one blinded and one non-blinded to study group. Analysis confirmed that the data were significantly paired, p<0.0001, and that the data was similar, p=0.1190.

**Biometric microglia classification:**

IBA-1 stains all microglia cells, both pro-inflammatory and anti-inflammatory. Davis et al. (2017) published the biometric cellular characteristics of these two microglia cell populations using an ImageJ technique.^9^ Microglia cells with pro-inflammatory characteristics have an average soma size of 85.9 μm^2^ and a roundness of 0.485. Microglia cells with anti-inflammatory characteristics have an average soma size of 42.5 μm^2^ and a roundness of 0.736. Our study classified IBA-1-positive cells into the two different behavior groups, following the biometric microglia characteristics as proposed by Davis et al. (2017).

**Lung injury score:**

Six NV subjects and six MV subjects had their left lungs harvested. Each lung had five standardized areas sampled, from the lung base to lung apex (see Diagram 1). The pulmonary tissues were sampled without inflation post-euthanasia, to preserve natural lung state. Immediately after harvesting the samples were submerged in formalin for further processing. An independent laboratory (Wax-it Histology Services Inc.), blinded to sample group, stained the slides with haematoxylin and eosin.


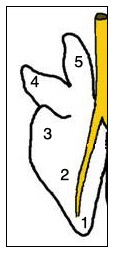


Diagram 1. Lung diagram showing the areas sampled-size, from the lung base to lung apex.

Blinded samples were scored for lung injury scoring method adapted from Matute-Bello score (Table 4).^10^ Septal thickness was not scored due to the tremendous degree of natural variation known to occur in this species regardless of any pathology. Each subject had twenty fields scored at 400X magnification. Each parameter was weighted according to Matute-Bello equation, as followed, score = [(20 X A) + (14 X B) + (7 X C) + (7 X D)]/(number of slides X100), to calculate the final score for each subject.

Table 4. Lung injury score parameters adapted from by Matute-Bello.

| **Parameters** | **Score per field** | | |
| --- | --- | --- | --- |
|  | 0 | 1 | 2 |
| A. Neutrophils in the alveolar space | none | 1 to 5 | >5 |
| B. Neutrophils in the interstitial space | none | 1 to 5 | >5 |
| C. Hyaline membranes | none | 1 | >1 |
| D. Proteinaceous debris filling the airspaces | none | 1 | >1 |

**Serum analysis:**

Baseline serum samples were taken during health checks one week prior to the experimental procedures. Sixteen pigs were available for blood sampling, and we elected to include all available samples in our analysis, totaling up to 16 serum samples analyzed in the NV group. Ten pigs, each of which was mechanically ventilated following the same experimental protocol, were available for blood sampling. We report results from all available samples in our analysis, totaling up to 10 serum samples analyzed for the MV group. Serum analyses were completed by an independent laboratory, Eve Technologies Corporation, which was blinded to study group for each sample, following ELISA protocols for immunoassays. Porcine Cytokine 13-Plex Discovery Assay was used to quantify TNF-α, IL-1α, IL-1β, IL-6, IL-8 and IL-10. Millipore Human S100B ELISA Assay was used for analysis of S100β. MyBioSource Pig Glial Fibrillary Acidic Protein (GFAP) ELISA was used for GFAP serum analysis, Porcine UCHL1 (Ubiquitin Carboxyl Terminal Hydrolase L1) Elisa for quantification of UCHL1, and Porcine Neuron Specific Enolase (Competitive) Elisa was used to quantify serum NSE concentration.

**Additional results (non-statistically significant):**

Hippocampal immunochemistry:

The percentage of IL-6-positive hippocampal cells was 4.12 median (3.36-4.31 IQR) in the NV group and 1.66 (0.00-3.97) in the MV group, p=0.1571. The percentage of TNF-α-positive hippocampal cells was 29.06 (18.38-30.57) in the NV group and 37.81 (29.18-48.01) in the MV group, p=0.1143.

Serum biomarkers:

NSE serum concentration was 32.23 ng/ml in the NV group (6.86-38.25) and 16.84 ng/ml in the MV group (5.66-24.28), p=0.0869. IL-1α serum concentration was 83.34 pg/ml (34.00-94.55) in the NV group and 13.65 pg/ml (6.13-60.96) in the MV group, p=0.0721. IL-6 serum concentration was 14.44 pg/ml (6.30-30.67) in the NV group and 37.67 pg/ml (21.12-453.50) in the MV group, p=0.1274. TNF-α serum concentration was 62.77 pg/ml (10.18-108.80) in the NV group and 15.02 pg/ml (0.00-28.25) in the MV group, p=0.0793.

**PaO_2_ /FiO_2_ ratio:**

Results reported as median and interquartile range. No statistically significant difference was found when compared the PaO_2_/FiO_2_ ratio of the NV group (568 mmHg; 546-571) to the PaO_2_/FiO_2_ ratio of the MV group in the start of the experiment (521 mmHg; 454-552, p=0.3700). The PaO_2_/FiO_2_ ratio of the MV group at the start of the experiment (521 mmHg; 454-552) was statistically different to PaO_2_/FiO_2_ ratio of MV group at the end of the experiment (403 mmHg; 357-444, p=0.0164) Individuals PaO_2_/FiO_2_ ratios (mmHg) were reported in the Table 3.

Table 4. PaO_2_/FiO_2_ ratio (mmHg)

| **NV group (mmHg)** | **MV group start (mmHg)** | **MV group end (mmHg)** |
| --- | --- | --- |
| 568 | 456 | 413 |
| 546 | 525 | 430 |
| 546 | 532 | 368 |
| 569 | 450 | 327 |
| 571 | 597 | 368 |
| 572 | 559 | 470 |
|  | 502 | 457 |
|  | 550 | 303 |
|  | 430 | 440 |
|  | 517 | 393 |

**References:**

1. Reynolds, S. C. *et al.* Mitigation of ventilator-induced diaphragm atrophy by transvenous phrenic nerve stimulation. *Am. J. Respir. Crit. Care Med.* **195**, 339–348 (2017).

2. Greene, S. A., Benson, G. J., Tranquilli, W. J. & Grimm, K. A. Effect of isoflurane, atracurium, fentanyl, and noxious stimulation on bispectral index in pigs. *Comp. Med.* **54**, 397–403 (2004).

3. Bassi, G. T., Rohrs, E., Fernandez, K., Ornowska, M. & Reynolds, C. S. Direct brain excision : An easier method to harvest the pig ’ s brain. *Interdiscip. Neurosurg.* **14**, 37–38 (2018).

4. Grasso, S. *et al.* ARDSnet ventilatory protocol and alveolar hyperinflation: Role of positive end-expiratory pressure. *Am. J. Respir. Crit. Care Med.* **176**, 761–767 (2007).

5. Haga, H. A., Tevik, A. & Moerch, H. Bispectral index as an indicator of anaesthetic depth during isoflurane anaesthesia in the pig. *Vet. Anaesth. Analg.* **26**, 3–7 (1999).

6. Kim, S. W., Roh, J. & Park, C. S. Immunohistochemistry for pathologists: Protocols, pitfalls, and tips. *J. Pathol. Transl. Med.* **50**, 411–418 (2016).

7. Holm, I. E. & West, M. J. Hippocampus of the domestic pig: A stereological study of subdivisional volumes and neuron numbers. *Hippocampus* **4**, 115–125 (1994).

8. Bassi, Thiago; Rohrs Elizabeth; Fernandez, Karl; Ornowska; Reynolds, S. An Easier Method to Analyze Stereologically the Pig’s Hippocampus. *J. neurosicence Neurol. Surg.* **4**, (2019).

9. Davis, B. M., Salinas-Navarro, M., Cordeiro, M. F., Moons, L. & Groef, L. De. Characterizing microglia activation: A spatial statistics approach to maximize information extraction. *Sci. Rep.* **7**, (2017).

10. Matute-Bello, G. *et al.* An official american thoracic society workshop report: Features and measurements of experimental acute lung injury in animals. in *American Journal of Respiratory Cell and Molecular Biology* **44**, 725–738 (2011).
